# Enriched Soil Organic Carbon Underpins Long‐Term Stable Crop Productivity Across China's Croplands

**DOI:** 10.1002/advs.78421

**Published:** 2026-10-22

**Authors:** Yuyang Huang, Yulin Shangguan, Zhongkui Luo, Peng Zhu, David Makowski, Zhenzhong Zeng, Qingxu Ma, Min Wei, Yuanyuan Du, Su Ye, Zhou Shi, Songchao Chen

**Affiliations:** ^1^ State Key Laboratory of Soil Pollution Control and Safety Zhejiang University Hangzhou China; ^2^ Zhejiang Key Laboratory of Agricultural Remote Sensing and Information Technology College of Environmental and Resource Sciences Zhejiang University Hangzhou China; ^3^ Department of Geography and Institute for Climate and Carbon Neutrality The University of Hong Kong Hong Kong China; ^4^ INRAE UMR 518 MIA‐PS AgroParisTech Université Paris‐Saclay Palaiseau France; ^5^ School of Environmental Science and Engineering Southern University of Science and Technology Shenzhen China; ^6^ Key Laboratory of Spectroscopy Sensing Ministry of Agriculture and Rural Affairs Zhejiang University Hangzhou China

**Keywords:** agricultural productivity, agriculture, crop yield, environmental science, food security, soil carbon sequestration, soil organic carbon stocks, sustainability

## Abstract

Maintaining stable agricultural production amid intensifying climate extremes is critical for global food security. While soil organic carbon (SOC) is a widely recognized pillar of agricultural sustainability, the extent to which SOC accrual can enhance crop stability remains poorly quantified. Here, comprehensive large‐scale datasets across China's maize and wheat croplands were leveraged to quantify nonlinear relationships using diverse nonparametric models. Critical SOC baselines (10.5–13.1 g kg^−1^ for maize; 8.4–9.9 g kg^−1^ for wheat) were identified as necessary for soils to effectively buffer crops against climatic fluctuations. Causal analysis revealed that approximately 20% of China's croplands were below these thresholds but exhibited significant marginal stabilizing effects from SOC enhancement. Specifically, in these high‐gains regions, a 1 g kg^−1^ increase in SOC improved crop stability by 6.2% to 11.1%, far exceeding the national average gains of 1.1% to 2.0%. Spatially, these pronounced gains were concentrated in municipalities characterized by high yields and extensive planting areas, highlighting that targeted SOC accrual in China's main breadbaskets was essential to secure food supply stability. By segmenting functional SOC ranges and spatially mapping marginal causal benefits, this study provides policymakers with quantitative evidence for targeted soil carbon management to enhance agricultural resilience.

## Graphical abstract

Quantifying the nonlinear relationships and causal effects of soil organic carbon (SOC) on crop stability reveals specific critical thresholds and saturation points. This partitions the national SOC spectrum into three functional phases. Integrating these spatial delineations with high‐resolution maps of marginal gains pinpoints regions with the greatest potential to enhance long‐term crop stability through targeted SOC sequestration.

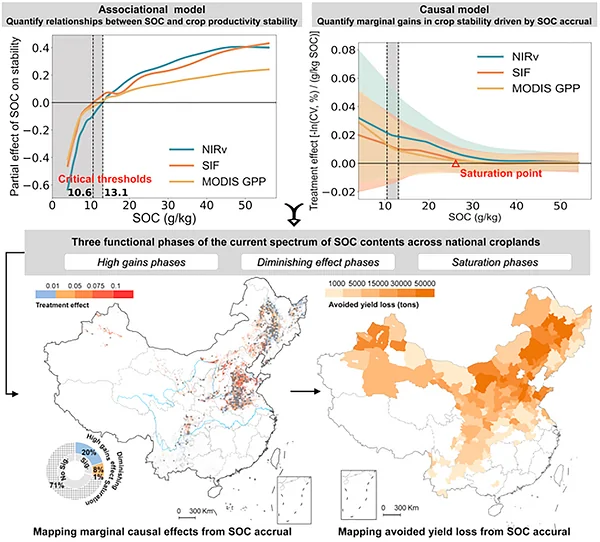

## 1 Introduction

Feeding a global population projected to reach 9 billion by mid‐century requires not only increasing total food production but also stabilizing the food supply [1]. While food security traditionally focuses on availability and accessibility, the stability dimension has become increasingly fragile under accelerating climate change [2]. Over recent decades, the rising frequency and intensity of extreme weather events have undermined the reliability of global food systems. Climate variability accounts for roughly one‐third of global crop production fluctuations, with a 1°C temperature increase projected to raise interannual yield variance by 7%–19% [3, 4]. Such instability propagates through supply chains, threatening livelihoods and market stability, and contributing to regional sociopolitical instability in extreme cases [5, 6]. Consequently, reducing interannual productivity fluctuations has emerged as a central challenge for resilient food systems [7].

Soils lie at the core of climate–plant productivity interactions, serving as a primary interface for the exchange of matter and energy within terrestrial ecosystems [8]. Soil organic carbon (SOC) is a key indicator of soil health and a fundamental component of the global carbon cycle [9, 10]. Increasing SOC stocks is therefore a cornerstone of cropland natural climate solutions, including cover cropping, reduced tillage, and organic amendments, which aim to jointly deliver climate mitigation and agricultural resilience [11, 12, 13]. By improving soil structure, water‐holding capacity, and nutrient cycling, higher SOC in cropland soils often enhances the buffering capacity of soils and supports crop physiological tolerance to environmental stress [14]. In this sense, SOC sequestration represents a dual benefit strategy that removes atmospheric carbon while strengthening the stability of crop production under climate variability.

Despite broad conceptual agreement on the stabilizing role of SOC, translating this understanding into quantitative and actionable evidence remains challenging. Three persistent knowledge gaps limit progress. First, traditional macroscopic studies inherently capture statistical correlations rather than direct causation [15, 16, 17]. At macro‐scales, SOC levels strongly co‐vary with climatic gradients and management practices. Previous regression models could not disentangle whether the observed stability was fundamentally driven by the biophysical nature of SOC or by concurrent human interventions. Second, isolating the direct causal contribution of SOC accrual from confounding influences is difficult in observational datasets [18, 19]. Without explicitly decoupling these complex confounding factors, the quantitative marginal benefit, specifically how much stability is gained from a 1 g kg^−1^ increase in SOC, remains a confounded correlative trend rather than a reliable metric. Third, although SOC is known to buffer crops against climate extremes [20, 21, 22], there is limited spatially explicit understanding of the environmental conditions under which this buffering effect is strongest based on previous macroscopic assessments. These uncertainties constrain the design of targeted and cost‐effective soil carbon management strategies, as policymakers lack quantitative metrics to balance sequestration costs against stability gains.

To address these gaps, we integrated a 20‐year remote sensing dataset (2001–2020) across China's maize and wheat croplands with complementary nonparametric statistical and causal inference approaches. China provides a compelling case study because its food system faces the dual pressures of maintaining stable production and reducing environmental impacts under intensifying climate change. We combined multiple independent proxies of crop productivity, including near‐infrared reflectance of vegetation (NIRv), solar‐induced chlorophyll fluorescence (SIF), and the MODIS gross primary productivity product (MODIS GPP), to construct robust proxies of annual growing‐season average crop productivity. Stability is subsequently defined as the negative natural logarithm of the coefficient of variation (CV), a metric that accounts for relative interannual variability of average productivity. The stability metrics were then analysed together with high‐resolution climatic, soil, and management variables within a two‐stage analytical framework. We first used nonparametric statistical models to characterize the relationship between cropland SOC content and productivity stability to identify critical baseline SOC thresholds. We then applied causal machine learning models to estimate the marginal gain in stability from increasing SOC while explicitly controlling for environmental and management confounders. Our analysis further partitions these gains into mean productivity enhancement and fluctuation reduction pathways and estimates the resilience dividend equivalent, providing a concrete measure of the yield secured by soil carbon enhancement. Ultimately, our study aims to move beyond generalized assumptions by identifying SOC levels that confer significant stability benefits for maize and wheat, quantifying the marginal stability gains associated with unit increases in SOC, and pinpointing the hotspots where SOC accrual delivers the greatest improvements in agricultural stability.

## 2 Results and Discussion

### 2.1 Critical Baseline SOC Thresholds in the Nonlinear Relationships

We characterize the annual average productivity of maize and wheat using three satellite‐derived proxies, NIRv, SIF, and MODIS GPP during the crops' key phenological stages, which capture the most critical period for dry matter accumulation. Based on these annual levels, we quantify crop stability using the negative natural logarithm of interannual CV (see Section 3). The stability metrics derived from the three proxies exhibit consistent spatial distributions and statistical characteristics (Figure 1a,b, Figure S1, Table S1). We identify regions with an interannual CV greater than 15% as having low stability, following the benchmark that typical crop production CV falls between 10% and 15% [23]. Spatially, such low stability of maize productivity is primarily observed in the high latitude regions of Northeast China and the arid and semi‐arid Northwest China. In contrast, regions with higher SOC, especially the core maize‐producing areas of Northeast China, demonstrate significantly higher stability (Figure S2). Wheat cultivation is concentrated in the North China Plain and shows lower overall stability than maize. Within this region, northern areas display greater interannual variability in wheat productivity, closely coinciding with lower SOC levels.

**FIGURE 1:**
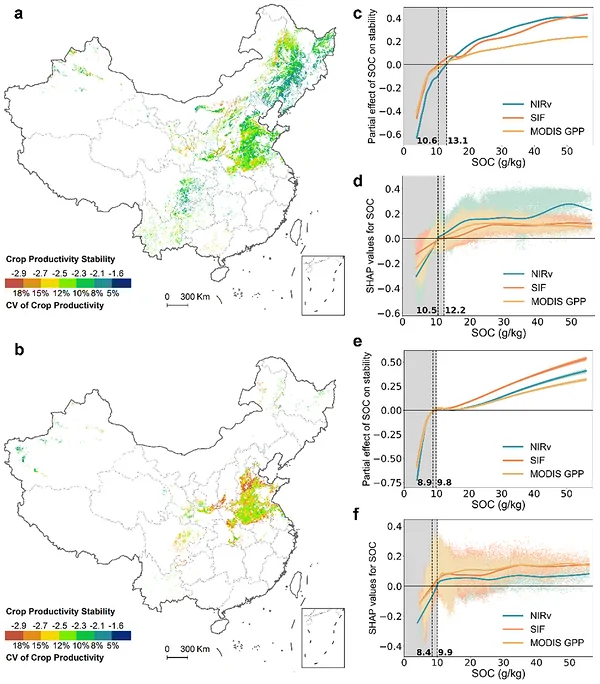
The relationships between SOC baselines and the stability of maize and wheat productivity. (a, b) Spatial pattern of the estimated stability of maize (a) and wheat (b) productivity. Stability is calculated as the negative logarithm of the coefficient of variation (CV) of crop productivity, where lower CV percentages indicate higher stability. (c, e) Relationships between baseline soil organic carbon (SOC) content and the stability of maize (c) and wheat (e) productivity, derived from Generalized Additive Mixed Models (GAMMs). (d, f) Relationships between SOC content and its SHapley Additive exPlanations (SHAP) values obtained from eXtreme Gradient Boosting (XGBoost) models predicting maize (d) and wheat (f) stability. In panels (c–f), crop productivity is evaluated using three independent proxies: near‐infrared reflectance of vegetation (NIRv, blue lines), solar‐induced chlorophyll fluorescence (SIF, orange lines), and MODIS gross primary productivity product (MODIS GPP, green lines) The vertical dashed lines and their corresponding values mark the critical threshold ranges for SOC content, which distinguish the positive and negative contributions of SOC to predict the crop productivity stability. Regions with SOC levels below the thresholds were predicted to have lower stability in maize and wheat productivity, while levels above the thresholds were associated with higher crop stability.

Generalized Additive Mixed Models (GAMM) and eXtreme Gradient Boosting (XGBoost) models consistently reveal a positive, nonlinear relationship between crop productivity stability and SOC levels across crop types and productivity proxies, with higher SOC levels invariably associated with greater stability (Figure 1c–f). Based on the zero‐crossing points of the fitted relationship curves, we identified critical SOC thresholds of 10.5 to 13.1 g kg^−1^ for maize and 8.4 to 9.9 g kg^−1^ for wheat across three different proxies and the two models. Regional analyses corroborated these findings (Figures S8 and S9). Although specific regional thresholds exhibit minor variations driven by distinct pedoclimatic conditions (higher in the Northeast and Northwest, and lower in the Huang‐Huai‐Hai Plain and Loess Plateau), they predominantly fall within the national bounds. Additionally, these satellite‐derived findings were also validated by in situ data from long‐term experimental sites, which still yielded similar critical thresholds based on actual crop yield and observed SOC (Figure S10). Above these thresholds, SOC contributes positively to model‐predicted stability. This reflects a positive statistical association within our predictive framework, where higher SOC levels are linked to increased stability in the model output. These critical thresholds correspond to moderate topsoil SOC levels, indicating that maintaining SOC above this baseline is essential for sustainable crop growth. Currently, only about 29.7% of maize and 3.4% of wheat croplands in China have SOC levels exceeding the upper bound of the critical threshold ranges. This indicates a substantial potential to enhance agricultural stability through SOC accumulation nationwide.

Importantly, the identified SOC critical thresholds represent a functional transition in the stabilizing role of SOC. To further substantiate this interpretation, we quantify the grid‐cell‐level sensitivity of productivity to annual mean temperature (*T_mean_*) and estimate the zone‐wide sensitivity of stability to temperature variability (*T_var_*) between regions below and above the critical SOC thresholds, defined as low SOC and high SOC zones. Sensitivity analyses confirmed that the following results remain consistent, regardless of the specific boundary values selected for the critical threshold ranges (Figures S6 and S7). The results indicate that grid‐cells belonging to low SOC zones exhibit generally higher absolute sensitivity to *T_mean_*. Specifically, average absolute sensitivity in these grid‐cells mostly exceeds 20%, implying that annual productivity can fluctuate by more than 20% per °C of temperature change. In contrast, in high SOC zones, the values remain notably lower, typically falling below 10% (Figure 2a–f, Table S6). This indicates that crop productivity in low‐carbon soils is more vulnerable to temperature fluctuations, regardless of whether the thermal effects are beneficial or detrimental. Likewise, the zonal sensitivity of stability to *T_var_* is more pronounced in low SOC zones. This confirms that the destabilizing impacts of climate variability are markedly stronger in these regions (Figure 2g–l), reinforcing the role of higher SOC as a critical buffer that moderates the influence of climate variability on the sustainability of crop growth.

**FIGURE 2:**
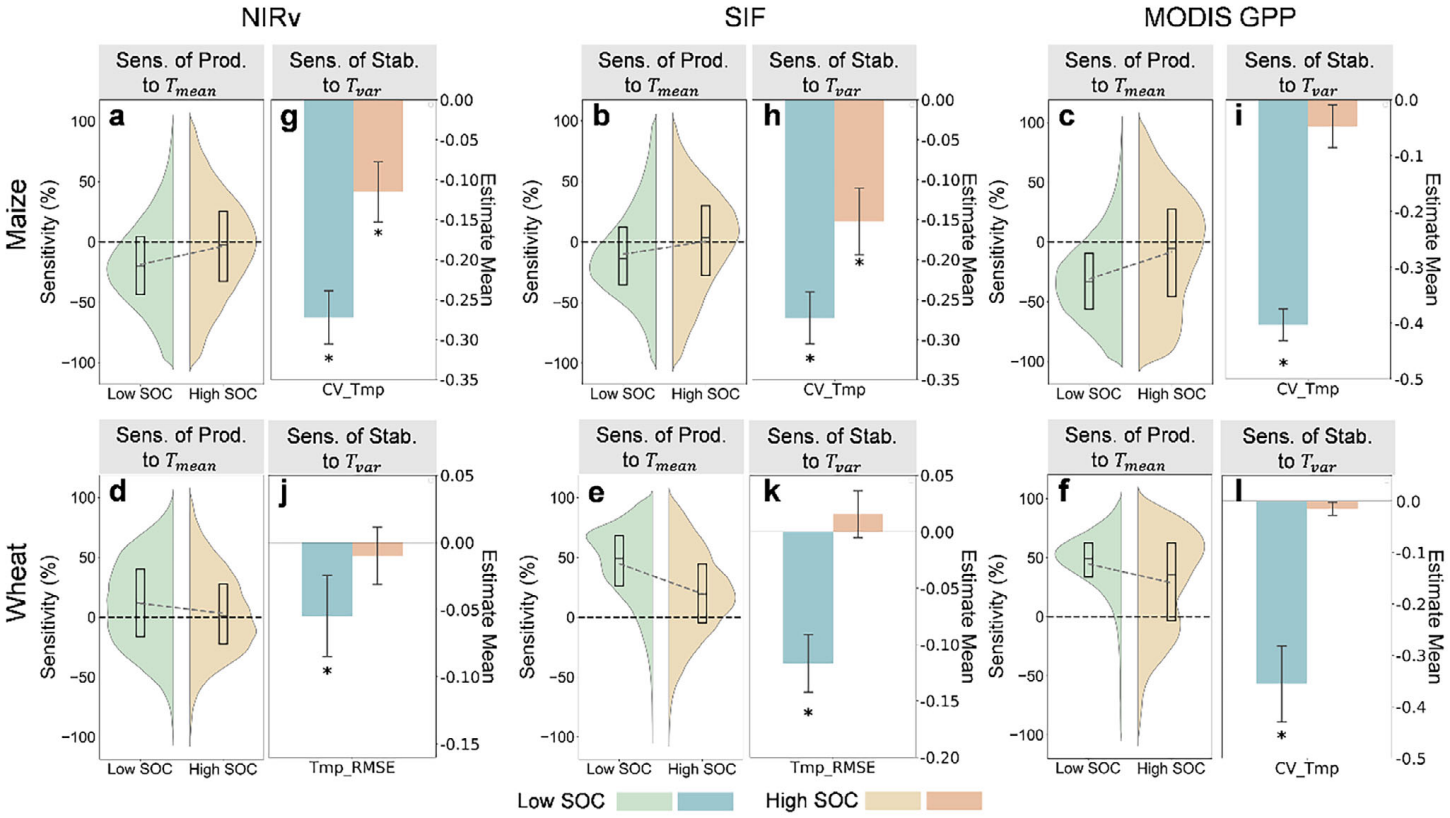
Crop productivity sensitivity to mean temperature and crop stability sensitivity to temperature variability in regions with SOC below and above critical thresholds. (a–f) Distributions of temperature sensitivity of crop productivity (Sens. of Prod. to T_mean_). Split‐violin plots illustrate sensitivities in low SOC and high SOC zones. The shaded violin envelopes represent the probability density distribution of the data. Inside the violins, the embedded box plots indicate the median and interquartile ranges. The closer the absolute sensitivity is to 0 (marked by the horizontal dashed black line), the less sensitive crop productivity is to annual temperature changes. The inclined dashed lines connect the mean values of the temperature sensitivities between the low and high SOC zones. (g–l) Sensitivity of crop stability to temperature variability (Sens. of Stab. to T_var_) in low SOC and high SOC zones. Bar charts display stability sensitivity in low SOC and high SOC zones. Negative mean estimates represent adverse effects of temperature variability on crop stability. The error bars represent the uncertainty (95% confidence intervals) derived from 100 bootstraps. The ^*^ indicates the coefficient is statistically significant at the 0.05 level. Low SOC zones are identified as regions with baseline SOC content below the lower limit of the SOC critical concentration range (10.5 g kg^−1^ for maize and 8.4 g kg^−1^ for wheat), while high SOC zones are regions with SOC content above the upper limit of the SOC critical concentration range (13.1 g kg^−1^ for maize and 9.9 g kg^−1^ for wheat).

Taken together, these results indicate that the identified critical SOC thresholds mark a transition of soil functional status. Below these levels, soils lack sufficient organic matter to sustain structural resilience, increasing vulnerability to climatic variability. Once cropland SOC levels exceed the thresholds, enhanced formation of stable macroaggregates and improved soil hydraulic retention promote a buffered root zone with more stable water and nutrient supply. This structural and functional maturation enables crops to maintain more consistent growth conditions under variable climatic stress, thereby strengthening the resilience of cropland productivity [24, 25].

### 2.2 Marginal Gains in Crop Productivity Stability Driven by SOC Accrual

Building upon the identified relationship between SOC content and crop productivity stability, we quantify the potential stability gains resulting from one‐unit (1 g kg^−1^) increase in SOC using the Causal Forest Double Machine Learning (CF‐DML) models. This dual framework is chosen for its ability to isolate SOC's effect from environmental confounders via orthogonalization, and to account for the spatial heterogeneity of its causal effects (see Section 3). At the national scale, the average treatment effects of SOC on crop stability are positive for both crops (Table S7), confirming a generally robust effect of SOC accrual in enhancing crop productivity stability. The estimated nonlinear dose‐response relationships (Figure 3c,g) further indicate that the stabilizing efficacy of SOC enhancement follows a pattern of diminishing marginal returns, whereby the marginal causal effect declines as baseline SOC increases and approaches saturation at approximately 26.1 g kg^−1^ for maize and 11.8 g kg^−1^ for wheat.

**FIGURE 3:**
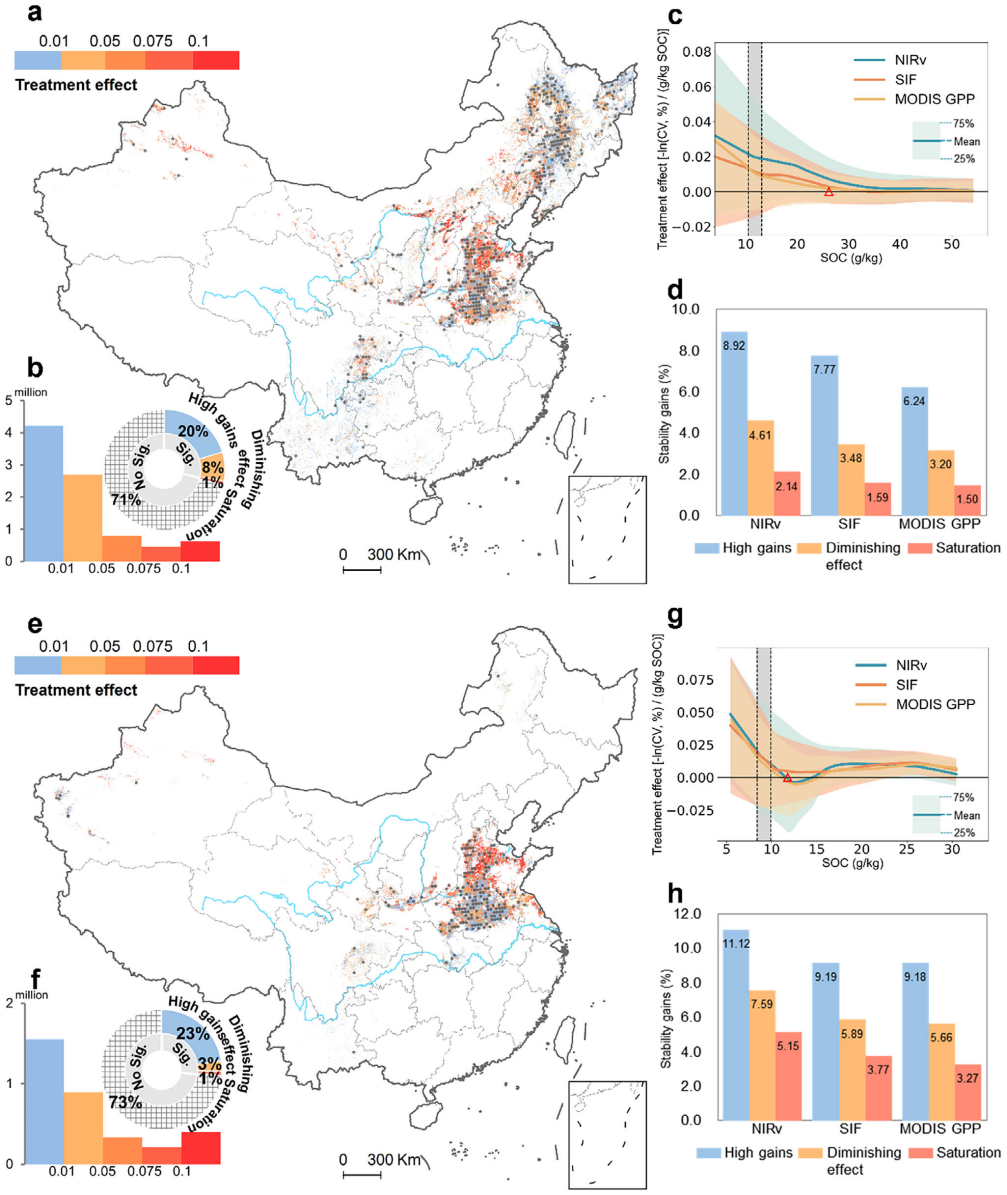
Treatment effect of increasing SOC on enhancing the stability of maize and wheat productivity. (a, e) The spatial distribution of the treatment effect of increasing SOC on enhancing the maize (a) and wheat (b) stability as estimated by the Causal Forest Double Machine Learning (CF‐DML) models. A lack of stippling indicates where at least 95 out of 100 bootstraps have an effect value greater than 0; in these areas, increasing unit SOC can significantly improve crop productivity stability. The maps and accompanying statistical charts (b, f) are based on the SIF proxy (results for the other proxies are in Figure S3). (b, f) The bar charts illustrate the number of grid‐cells categorized by the magnitude of the SOC treatment effect shown in the colour legend, and the pie charts display the proportion of grid‐cells exhibiting a statistically significant (Sig.) vs. non‐significant (No Sig.) SOC effect, further broken down by their distribution across different SOC functional stages (High‐gains, Diminishing effect, and Saturation). (c, g) Dose‐response relationships between baseline SOC content and its marginal causal effect on maize (c) and wheat (g) stability. Solid lines represent the mean values estimated by the CF‐DML models from 100 bootstraps. The shaded areas represent the interquartile range (25th–75th percentiles) of the estimated causal effects. The vertical dashed lines indicate the ranges of the critical SOC thresholds. The triangle sign indicates the saturation SOC values for improving stability by increasing SOC. (d, h) The bar charts show the average stability gains across three SOC functional stages in maize (d) and wheat (h) croplands. High‐gains phase: SOC ≤ 13.1 g kg^−1^ (maize) and ≤ 9.9 g kg^−1^ (wheat). Diminishing effect phase: 13.1 < SOC ≤ 26.1 g kg^−1^ (maize) and 9.9 < SOC ≤ 11.8 g kg^−1^ (wheat). Saturation phase: SOC > 26.1 g kg^−1^ (maize) and > 11.8 g kg^−1^ (wheat).

To ensure robustness, the dose‐response curves were averaged across 100 bootstrap iterations and further supported by rigorous refutation tests (Table S5, see Section 3). We defined significant effect grid‐cells as those for which more than 95% of bootstrap models yielded positive causal effects. At the national scale, a 1 g kg^−1^ increase in SOC reduces the CV of crop productivity by 1.11 to 1.97% on average across different crop types and productivity proxies, while within significant effect zones, this stability gain is markedly larger, ranging from 4.59% to 9.15% (Table S7). Spatially, areas with non‐significant effects are widely dispersed, showing subtle stabilizing responses to a 1 g kg^−1^ SOC increase. However, grid‐cells with significant treatment effects are highly clustered. These critical hotspots are primarily localized in northern and northwestern China, corresponding to areas with fundamentally low baseline SOC levels (Figure 3a,e).

Identifying the critical SOC threshold aims to determine the minimum baseline required to buffer crops against climatic shocks. Sufficient SOC provides essential organic binding agents that form stable macroaggregates, thereby enhancing soil water and nutrient retention. The saturation concentration correlates with the diminishing marginal benefits of carbon enrichment on crop stability. As SOC increases, soil porosity, water‐holding capacity, and the surface area of reactive minerals eventually approach their maximum physical capacities. Consequently, these marginal benefits gradually diminish toward zero. Based on the two pivotal points, the current spectrum of SOC contents across national croplands can be categorized into three distinct functional phases. First, the high‐gains phase occurs in grid cells where SOC levels are below the upper bound of the critical thresholds and exhibit significant stabilizing effects. Approximately 30% of maize and wheat croplands nationwide show significant SOC‐induced stabilizing effects, the majority of which, representing about 20% of the national total, fall into the high‐gains phase (Figure 3b,f, Figure S3). A sensitivity analysis utilizing the lower threshold bound yields a conservative estimate of the spatial extent of the high‐gains phase, with coverage remaining robustly above 15% (Table S9). In these high‐gains areas, a 1 g kg^−1^ increase in SOC yields stability gains ranging from 6.24% to 11.12% across different crop types and productivity proxies (Figure 3d,h). Previous studies have also similarly reported larger crop production benefits from SOC enhancement when baseline SOC is below 10–12 g kg^−1^ [12, 26]. Second, the diminishing effect phase spans the range between the critical thresholds and the saturation concentrations, where additional SOC accumulation continues to stabilize crop productivity but with progressively smaller stability gains. Finally, the saturation phase is reached above the saturation concentrations, where marginal causal effects of SOC enhancement approach zero. Aggregating to the municipal scale, cities ranking in the top 30% for high‐gains grid‐cell coverage across all productivity proxies are classified as high‐gains cities (Figure 5a,b). These cities are predominantly clustered in north‐central China for maize and across the North China Plain for wheat.

The broad interquartile ranges surrounding the dose‐response curves (Figure 3c,g) further indicate that the marginal stabilizing effect of SOC is not determined solely by baseline SOC levels but is strongly modulated by local environmental conditions. By stratifying primary environmental drivers in conjunction with baseline SOC levels (Figure 4), we show that the stabilizing effect of increasing SOC is amplified under specific stressors. For maize, SOC benefits are enhanced under alkaline soil conditions and greater precipitation variability. For wheat, SOC effects are stronger in areas with higher silt content, lower precipitation, and more rapid warming trends. These settings represent pronounced climatic variability and soils prone to structural instability and nutrient constraints, because higher silt content increases soil vulnerability to compaction and crusting, and alkaline conditions often limit nutrient availability. Mechanistically, these patterns are consistent with SOC‐mediated regulation of nutrient bioavailability and soil structure. Specifically, organic acids produced during SOC decomposition can increase nutrient accessibility through chelation [27, 28], and higher SOC levels can reduce soil compactibility and increase elasticity by promoting the formation of stable macroaggregates [24, 29]. The structural improvement also generates a sponge effect, enhancing water retention and air permeability, thereby buffering crops against greater climatic variability [20, 21, 30].

**FIGURE 4:**
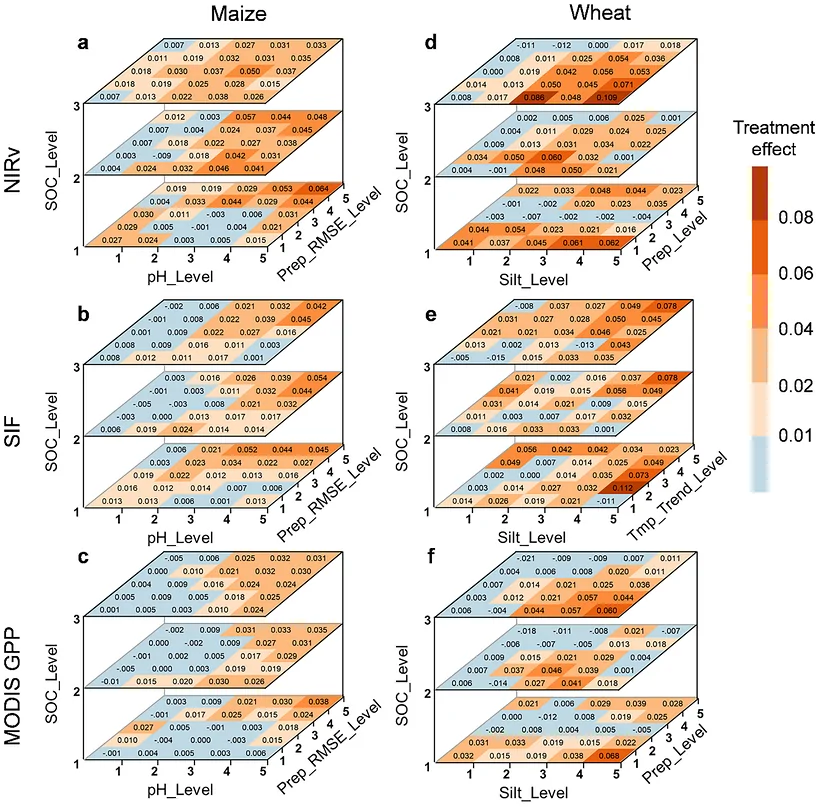
Distribution of SOC treatment effects across dominant edaphoclimatic gradients and baseline SOC levels. The 3D stacked grid planes display the interactive influence of dominant variables on the SOC treatment effect for maize and wheat. The rows correspond to three distinct crop productivity proxies used for the evaluation. For each plane, the vertical axis categorizes baseline SOC into three discrete levels representing functional stages of SOC intervention (Level 1: High‐gains Phase; Level 2: Diminishing effect Phase; Level 3: Saturation Phase). The horizontal axes represent the selected variables that exhibited the highest correlation coefficients with SOC causal effects from each model (see Figure S4). Within each SOC level, these variables are divided into five quantile levels (1–5). For soil pH, which represents a gradient from acidic to alkaline, the ranges are 1 (4.39–6.73), 2 (6.73–7.27), 3 (7.27–7.69), 4 (7.69–8.00), and 5 (8.00–9.12); for other variables, levels 1 to 5 denote a progressive increase in magnitude. The colour gradient of each grid represents the average SOC treatment effect within specific multifactorial levels; numerical annotations within each cell indicate the exact average treatment effect values.

Collectively, increasing cropland SOC acts as a critical compensatory strategy that strengthens the resilience of agricultural systems to external shocks, especially under conditions of physicochemical soil constraints and heightened climatic stress. Geographically, the strongest marginal stability gains for both maize and wheat are concentrated in Northwest China and the northern part of the North China Plain. These regions are frequently exposed to extreme thermal and hydrological variability and therefore exhibit higher stability gains from per unit increase in SOC. As climate anomalies increasingly become the norm, soil carbon sequestration strategies should be tailored according to local environmental conditions. Spatially targeted soil carbon management that prioritizes vulnerable agricultural systems with restrictive pedoclimatic conditions will allow policymakers to fully exploit the stability dividend of soil carbon and reinforce food security.

### 2.3 Regional Carbon Sequestration Strategies to Achieve Stability Goals

To contextualize the macro‐level implications for food security, we translated the grid‐cell‐level stability gains from SOC accrual into a municipal‐level resilience dividend equivalent. By defining a typical external agricultural shock as a one‐standard‐deviation yield penalty, we estimate the resilience dividend equivalent as the avoided yield loss attributable to a 1 g kg^−1^ increase in SOC (see Section 3). Before calculating this indicator, since the stability metric in this study is formulated as the negative natural logarithm of CV, we can mathematically partition the overall SOC‐driven stability gains into two complementary pathways, including the enhancement of mean productivity and the reduction of absolute interannual variability. To achieve this decomposition, we estimate the treatment effect of SOC on the natural logarithm of mean productivity using analogous causal models (see Section 3), enabling us to quantify the proportional contributions of these two functional components (Figure 5c,d). At the national scale, our causal framework reveals that the primary driver of stabilization is the productivity enhancement pathway, which accounts for 62.6% in maize croplands and 74.7% in wheat croplands of the total SOC‐induced stability gain. Concurrently, the remaining 37.4% in maize and 25.3% in wheat of the stability dividend originates directly from the variance‐reduction pathway. Therefore, independent of the mean productivity increases, SOC also plays an important role in suppressing the absolute magnitude of long‐term productivity fluctuations.

**FIGURE 5:**
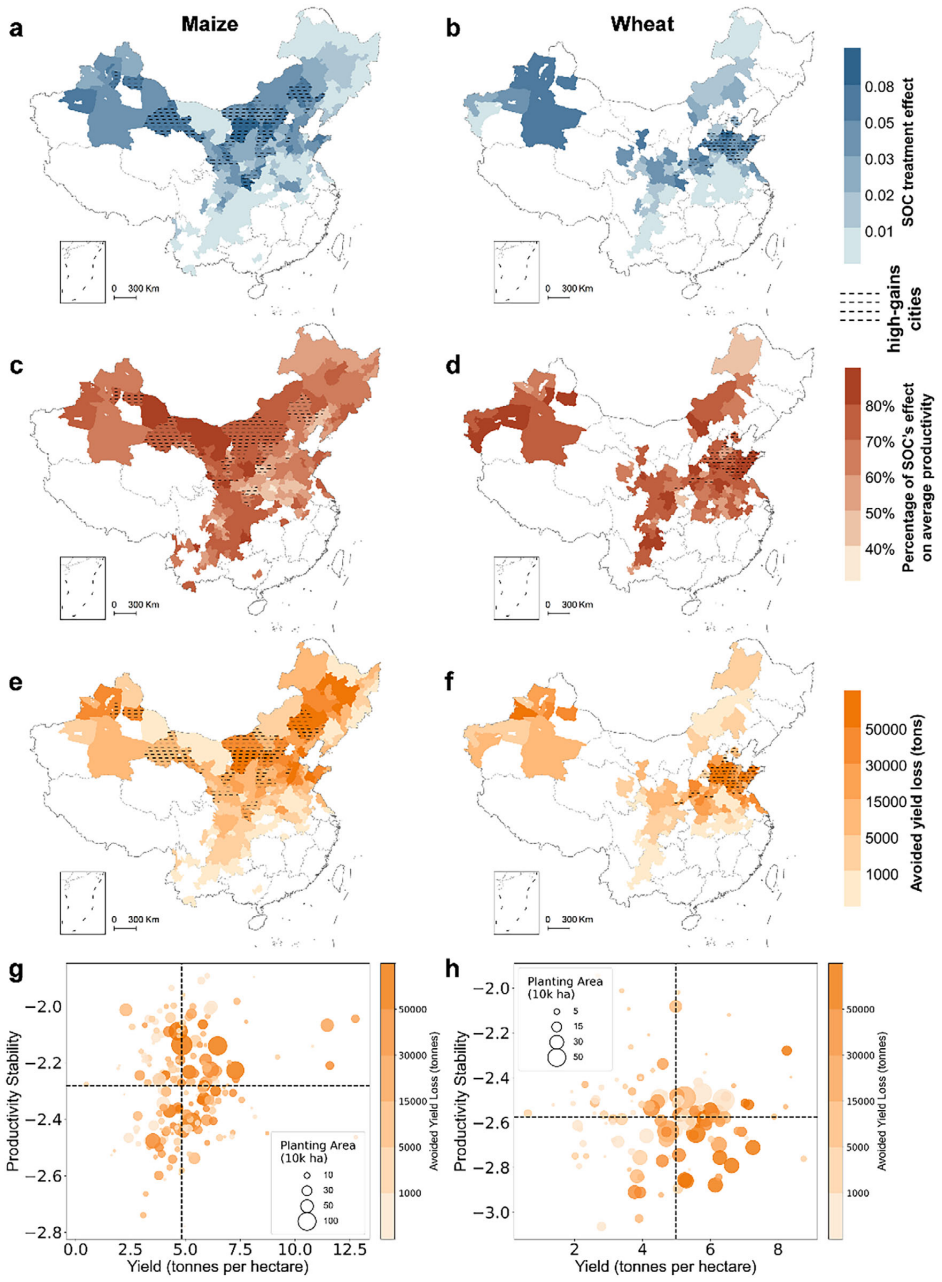
Quantifying the avoided crop yield loss attributable to a 1 g kg^−1^ increase in SOC at the municipal level. (a, b) Spatial maps show the municipal‐level average of the treatment effect of SOC on maize (a) and wheat (b) productivity stability. (c, d) Maps show the municipal‐level average of the percentage of SOC's effect on improving mean productivity for maize (c) and wheat (d). (e, f) Maps show the estimated SOC‐driven avoided crop yield loss during external shock years. External shock years are defined as those experiencing a one‐standard‐deviation yield penalty. The dashed patterned areas overlaid on the maps refer to identified high‐gains cities. (g, h) Quadrant analysis of crop yield and productivity stability across municipalities and the quadrant distribution of SOC‐driven avoided yield loss. The orange colour gradient of the scatter points reflects the avoided yield loss attributable to a 1 g kg^−1^ SOC increase (see Section 3). The size of each point is proportional to the crop planting area. The vertical and horizontal dashed lines represent the median values of crop yield and productivity stability, respectively, dividing the scatter plot into four scenarios: the top‐right quadrant indicates ‘high yield and high stability’, the bottom‐right indicates ‘high yield and low stability’, the top‐left indicates ‘low yield and high stability’, and the bottom‐left indicates ‘low yield and low stability’.

Integrating municipal crop yield records with the SOC‐driven stability gains and the decomposed stability pathways, we quantify the municipal‐level resilience dividend equivalent during typical agricultural shocks (−1σ yield anomalies) (see Section 3). Our results reveal that a 1 g kg^−1^ SOC accrual secures an average of 13 000 tonnes of crop yield per municipality during shock years, cumulatively averting approximately 2.6 and 1.7 million tonnes of production losses nationally, respectively. But the spatial distribution of this dividend is highly non‐uniform (Figure 5e,f). In identified high‐gains cities, the resilience dividend per unit of SOC increase is nearly three to four times the national average. Remarkably, although these approximately 30 cities represent only a small fraction of China's total cropland area, their collective contribution exceeds one‐third of the total national resilience potential (Table S10), highlighting the high efficiency of spatially targeted interventions.

By categorizing municipalities based on their 20‐year average yield and stability, we observe that numerous municipalities with extensive crop planting areas and high crop yields exhibit larger resilience dividend equivalents (Figure 5g,h). These regions often operate under intensive management with high input levels, yet they remain fundamentally vulnerable to climate‐induced variability. Many of the identified high‐gains cities currently operate in a state of ‘high yield and low stability’, where average yields exceed the national median but stability remains below it. Therefore, although these regions constitute the nation's core breadbaskets, there still remains large potential to improve the stability of long‐term food supply.

Our findings suggest that the observed stability gap in these breadbaskets is primarily driven by SOC levels remaining below the critical thresholds required for optimal hydrothermal regulation and limited pedoclimatic conditions. Consequently, prioritizing SOC accrual in the high‐gains regions offers a high‐leverage strategy for climate adaptation. Moving beyond broad‐based carbon sequestration policies, targeted management focusing on these hotspots can maximize the efficiency of soil‐based climate mitigation and ensure a more robust national food supply in an increasingly volatile climate.

### 2.4 Conclusions and Implications

Quantifying the stabilizing effect of SOC is essential for advancing carbon sequestration strategies and strengthening agricultural resilience. Here, we provide large‐scale, quantitative evidence that SOC enrichment buffers long‐term fluctuations in crop productivity through nonlinear pathways. We identify critical SOC baseline thresholds required to activate the stabilizing benefits, indicating that regions with SOC levels below the thresholds possess substantial potential for resilience improvement through SOC accumulation. Our casual analysis demonstrates that while a unit increase in SOC generally enhances crop stability, this causal benefit is non‐uniform. Specifically, areas characterized by low SOC baselines and restrictive pedoclimatic conditions show significantly more pronounced stabilizing effects. These high‐gains areas account for approximately 20% of China's cropland; here, a 1 g kg^−1^ increase in SOC improves crop stability by 6.2% to 11.1%, compared to a mere 1.1% to 2.0% at the national average. At the municipal level, this 1 g kg^−1^ SOC accrual mitigates shock‐induced yield losses by an average of 13 000 tonnes per municipality. But in identified high‐gains cities, the averted yield loss is up to three to four times higher. Furthermore, many of these cities are characterized by low stability, high yield, and large crop planting areas. Therefore, targeted SOC accrual management in these core breadbaskets is vital for narrowing crop stability gaps and safeguarding a sustainable national food supply.

Despite these insights, certain limitations and sources of uncertainty remain. First, inherent discrepancies in spatial resolution among gridded variables may introduce noise when precisely delineating critical SOC thresholds. However, our multiscale sensitivity analyses (Figure S5) reveal that the identified critical SOC thresholds are robust to spatial aggregation. While coarser resolutions tend to compress variable variance and underestimate the magnitude of localized causal effects, the overall stabilizing effect remains consistent. Meanwhile, to mitigate these data‐driven uncertainties and ensure robustness, we validated our findings across multi‐million‐point datasets using multiple productivity proxies and nonparametric statistical models. The observed strong consistency suggests that our high‐resolution framework accurately captures the SOC‐stability relationships, and any residual resolution mismatches (e.g., coarse management variables) likely result in a conservative underestimation of the true SOC buffering effect.

Second, while the CFDML models effectively quantify the marginal stabilizing benefits of SOC accruement, observational causal inference fundamentally relies on the assumption of unconfoundedness. Although we have incorporated a comprehensive set of agricultural practice datasets to the limits of current data availability, our estimates cannot entirely rule out the influence of unmeasured local management practices. Indeed, our sensitivity analysis explicitly reveals that omitting management variables causes the marginal causal effect of SOC to be overestimated, as the stabilizing benefits from anthropogenic interventions are erroneously attributed to the soil (Tables S7 and S8). Consequently, unmeasured confounding factors could still contribute to a potential overestimation of our derived causal effects. To mitigate these uncertainties, we conduct multiple refutation tests and 100 bootstrap iterations to ensure the robustness and causal validity of our findings. The parallel experiments across different productivity proxies minimize the potential bias from unmeasured management practices, as different proxies are characterized by distinct sensitivities to various sources of interference. Moreover, a purely statistical framework cannot mechanistically decouple the complex biophysical benefits of SOC from its biogeochemical nutrient‐supply effects. Future research should therefore integrate our empirical findings with process‐based agricultural simulators, particularly under future climate change scenarios, to explicitly trace the temporal dynamics of SOC‐climate interactions and fully explain the physiological mechanisms driving crop resilience.

Beyond the scientific framework, our findings hold profound practical implications. Although this analysis focuses on China, our analytical logic is broadly applicable to agroecosystems worldwide. Specifically, by quantifying critical thresholds to partition the SOC ranges into distinct functional phases, and integrating these with high‐resolution marginal gain maps, this framework can precisely pinpoint croplands with the highest potential for stability improvement from soil carbon input management. Our study is not designed to merely reconfirm the qualitative consensus that SOC can improve the stability of crop production. Instead, we demonstrate that the stabilizing efficacy of SOC enhancement is modulated by baseline SOC levels and the local environmental context. This context‐dependent and spatially heterogeneous paradigm challenges the conventional macroscopic correlative assumptions. It provides a quantitative basis for policymakers to shift from uniform agricultural subsidies to spatially targeted, climate‐smart interventions. By prioritizing soil carbon sequestration efforts and financial incentives in the high‐gains regions, global agricultural policies can maximize the resilience dividend and cost‐effectively secure food systems against climate volatility.

## 3 Materials and Methods

### 3.1 Materials

#### 3.1.1 Cropping Area and Phenology Data

In this study, the maize cropping area was sourced from the China Crop Dataset–Maize at a 30‐m resolution [31], and we identified grid cells where maize was consistently cultivated each year from 2001 to 2020. The wheat cropping area was derived from the dataset of main grain land changes in China over 1985–2020 [32], also at a 30‐m resolution. The regions of single‐season wheat, wheat‐maize rotation, and wheat‐rice rotation in 2020 were aggregated to determine the distribution of wheat cultivation across China. The dataset of ChinaCropPhen1km [33] (available for 2001–2019) was employed to compute the multi‐year average Julian Day of the Year for key phenological stages of maize and wheat. We specifically delineated the active growing season from the green‐up date (for wheat) and the V3 stage (for maize) through to the maturity date because these specific phenological windows are the critical vegetative and reproductive growth phases for determining final biomass and grain yield. By focusing on these critical stages, we also minimized the spectral noise from soil background during early growth and senescence. Finally, the spatial extent of maize and wheat distribution in China can be seen in Figure S1.

#### 3.1.2 Crop Productivity Data

To ensure the robustness of our assessment and mitigate uncertainties associated with individual remote sensing products, we employed three widely validated remote sensing proxies to represent maize and wheat productivity, including NIRv, SIF, and MODIS GPP. These three proxies were selected because they represent crop productivity through different mechanisms, including structural (NIRv), physiological (SIF), and algorithmic (MODIS GPP). Annual crop productivity was quantified by averaging monthly remote sensing metrics during the key phenological stages (from V3 to maturity for maize and from green‐up to maturity for wheat). The averaging approach serves as a robust proxy for the seasonal integrated primary production, as it accounts for both the intensity and the duration of carbon assimilation. Therefore, these metrics are able to capture the annual variability of crop productivity and to estimate agricultural stability.

NIRv, a new vegetation index, was defined as the product of the normalized difference vegetation index (NDVI) and near‐infrared reflectance (NIR), representing the proportion of pixel reflectance attributable to the vegetation in the pixel [34]. Badgley et al. [35] compared NIRv with site‐scale FLUXNET GPP estimates, revealing high correlation between NIRv and monthly or yearly GPP; therefore NIRv can be utilized to quantify vegetation productivity. The satellite reflectance data were derived from the MOD09Q1 product (8‐day composite, 250‐m resolution). After cloud removal and crop area masking in Google Earth Engine, we calculated the monthly median NDVI and NIR values from 2001 to 2020. We then computed monthly NIRv:

(1) $$\mathrm{NIRv}\;=\;\mathrm{NIR}\cdot \left(\frac{NIR-RED}{NIR+RED}-0.08\right)$$

where NIR and RED are near‐infrared reflectance and red reflectance, respectively. Subtracting 0.08 from NDVI is an attempt to partially account for the NDVI of bare soil. Subsequently, we averaged monthly NIRv data during the above key phenological stages and use the resulting values as proxies for maize and wheat productivity.

SIF, as a direct physiological probe of photosynthetic rates, has been acclaimed as a proxy for crop productivity in recent years [36]. Here, we employed high‐resolution SIF data for China (HCSIF, 8‐day composite, 500‐m resolution) to quantify multiyear monthly median crop productivity. Then, we repeated the same process as for NIRv to compute annual maize and wheat productivity from 2001 to 2020.

The standard MODIS GPP products, integrating MODIS remote sensing data and meteorological inputs with the semi‐empirical light use efficiency model to estimate gross primary productivity of global vegetation [37], are currently the most widely used GPP product. In this study, the MODIS GPP dataset was obtained from MOD17A2H.006 Terra Gross Primary Productivity product with 500 m spatial resolution and 8‐day temporal resolution on Google Earth Engine. We selected data from “Gpp” band, spanning from 2001 to 2020 and computed monthly median values within maize and wheat cropping areas. Subsequently, we averaged the monthly GPP composite values during the key phenological stages to obtain proxies for the annual maize and wheat productivity.

#### 3.1.3 Soil Data

The content of soil organic carbon (SOC) and other soil property data at 250 m spatial resolution were all sourced from High‐Resolution National Soil Information Grids of China [38]. The soil survey samples used to generate this dataset span from 2010 to 2018. Given that soil properties evolve relatively slowly over time, we consider this dataset suitable for our study period. The soil variables were extracted from the dataset, including SOC content, bulk density (BD), pH, clay content, silt content, sand content, and cation exchange capacity (CEC), at a resolution of 250 m for the 0–5, 5–15, and 15–30 cm depth layers. The values of each soil property were calculated for each grid cell. We used a soil layer depth weighting method to standardize the soil data into a 0–30 cm depth interval, and obtained the soil covariates (Table S2) for fitting crop productivity stability models shown below.

#### 3.1.4 Meteorological Data

Daily meteorological data to derive climatic covariates were sourced from the high‐resolution (1 km) and long‐term (available for 2001–2019) gridded dataset for temperature and precipitation across China (HRLT) [39]. Based on the HRLT dataset, we computed the annual average temperature and precipitation over the growing seasons for maize and wheat, respectively, and averaged the annual data across all years to obtain the mean temperature and precipitation in each grid cell. Additionally, the coefficient of variation for both annual temperature and precipitation was computed to represent the interannual variability of temperature and precipitation in each grid cell.

We also computed the annual trends of mean temperature and total precipitation during crop growing periods. First, to eliminate the climatic seasonality, we converted the monthly composite temperature and precipitation into standardized anomalies:

(2) $$\Delta_{X}\;\left(i,t\right)=\frac{X\left(i,t\right)-\mu_{X}\left(i,m\right)}{\sigma_{X}\left(i,m\right)}\;,t\in m$$

where Δ_*X*_(i,t) represents the standardized anomalies; *X*(*i*, *t*) denotes the value of grid‐cell *i* at time *t* (monthly time step); μ_*X*_(*i*,*m*) and σ_*X*_(*i*,*m*) represent the long‐term mean and standard deviation of grid‐cell *i* for the corresponding calendar month m across all years of the dataset.

Subsequently, we employed linear regression models to estimate annual trends of temperature and precipitation. The slopes, denoted as β, were extracted as the trends for standardized climate variables (expressed in σ/year):

(3) $$\Delta_{X}\;\left(i,t\right)=\alpha_{i}+\beta_{i}\times t+\varepsilon_{\left(i,t\right)}$$

where Δ_*X*_(i,t) represents the standardized anomalies of temperature or precipitation for grid‐cell *i* across year *t*; β_*i*_ is used to quantify the annual trends of climatic variables in grid‐cell *i*.

Additionally, the residuals of temperature and precipitation annual trends were also computed to represent the climatic variability, as these residuals effectively decouple interannual deviation from long‐term trends. Here, we employed the root mean square error (RMSE) to quantify residuals of temperature and precipitation trends:

(4) $$\mathrm{RMSE}\;=\sqrt{\frac{\sum_{i=1}^{n}{\left({\hat{y}}_{i,t}-y_{i,t}\right)}^{2}}{n}}$$

where y_i,t_ denotes the standardized anomaly value of temperature or precipitation in grid‐cell *i* (computed from Equation 2), ${\hat{y}}_{i,t}$ denotes the predicted value derived from the linear model (computed from Equation 3), and *n* denotes the sample size.

For each grid‐cell, the effect of temperature and precipitation was summarized by μ_*X*_ (mean), β_*X*_ (trend), *RMSE_X_* and *CV_X_*. All these climatic covariates were resampled to 250 m resolution using the nearest neighbour method (Table S2).

#### 3.1.5 Management Data

To represent anthropogenic inputs and management practices across China's croplands, we incorporated six key variables: nitrogen (N) and phosphorus (P) fertilizer use, the proportion of irrigated cropland, the irrigation water use (IWU), changes in planting density, and tillage practices. Specifically, the spatial distribution of N fertilizer use was derived by averaging annual data (2001–2018) from a 5‐km historical dataset [40]. Similarly, P application rates for wheat and maize were obtained by averaging crop‐specific 1‐km records from 2004 to 2016 [41]. The fraction of irrigated cropland was calculated as the multi‐year average of annual 250‐m maps (2001–2020) sourced from the CIrrMap250 dataset [42]. The estimated long‐term average IWU data were obtained from the annual 500m Irrigation Water Use dataset in China from 2004 to 2019 [43]. Lacking high‐resolution planting density records, we calculated the linear trend of the annual maximum Leaf Area Index (LAI) from 2001 to 2020 (using the 500‐m MOD15A2H product) to serve as a proxy for planting density changes [44]. Data on tillage practices were extracted for China from a global gridded dataset [45] and reclassified as categorical variables. Finally, all management variables were resampled to a 250‐m resolution using nearest‐neighbour interpolation to ensure spatial consistency with the other covariates and target variables.

### 3.2 Data Analysis Methods

#### 3.2.1 Characterizing Crop Productivity Stability

For the assessment of crop productivity stability, statistical dispersion metrics, including standard deviation and the coefficient of variation (CV), were frequently employed to quantify long‐term fluctuations in crop productivity [16, 22]. However, due to influences such as climate change, management practices, and technological advancements, it is necessary to detrend the average annual crop productivity over the period 2001–2020, thereby focusing on the year‐to‐year fluctuations in crop productivity. Therefore, we computed the variability of crop productivity in each grid cell as:

(5) $$CV_{detrend}\;\left(\%\right)=\frac{StdDev\left(residuals\right)}{Mean\left(productivity\right)}\;\times 100$$

where *StdDev*(*residuals*) represents the standard deviation of the residuals derived from a linear regression of annual crop productivity from 2001 to 2020 in each grid cell, and *Mean*(*productivity*) represents the average annual crop productivity over the 20‐year period in each grid cell. In order to ensure normality and facilitate interpretation, we then transformed the CV using the negative natural logarithm ($-\ln(CV_{detrend})$). This transformed index served as a metric for crop productivity stability, where a higher value indicated greater stability of crop productivity. There were totally above 8.8 million valid grid cells in the maize cropping area and nearly 3.4 million in wheat cropping area.

#### 3.2.2 Identifying the Threshold Between SOC and Crop Productivity Stability

Two different models were utilized separately to evaluate non‐linear relationships between SOC and crop productivity stability. First, we employed the generalized additive mixed model (GAMM) because it allowed for flexible smoothing functions to characterize complex ecological relationships and random effects to account for inherent spatial autocorrelation [46]. The model was implemented using the ‘gam’ function of the ‘mgcv’ package in R, formulated as follows:

(6) $$S_{i}=\beta_{0}+s_{1}\left(\log\left(SOC_{i}\right)\right)+\sum_{k=1}^{K}s_{k}\left(X_{ki}\right)+s_{tp}\left(lon_{i},lat_{i}\right)+\varepsilon_{i}$$

where *S_i_* is the stability metric (− ln (*CV_detrend_*)) for grid‐cell *i*; s_1_(·) is the smooth function for SOC; ∑*s_k_* (*X_ki_*) represents the additive smooth functions for control covariates *X*, including soil, climatic, and management variables; *s_tp_* (*lon_i_*,*lat_i_*) denotes the two‐dimensional thin‐plate spline function of longitude and latitude coordinates to account for spatial autocorrelation; β_0_ represents the intercept, and ε_*i*_ is the residual error term. Notably, we assessed multicollinearity among covariates using the variance inflation factor (VIF) and the silt content was excluded from the model construction as its VIF value exceeded the threshold of 10. Additionally, to address the skewed distribution of SOC data, a logarithmic transformation was applied prior to modelling. The model accuracy can be seen in Table S3. The positive fitted values of the smoothing function for SOC represented its positive contribution to the modelled crop stability, and vice versa. Therefore, we defined the critical SOC threshold as the zero‐crossing point where the first instance shifts from negative to positive. Surpassing this threshold indicates that baseline SOC content above this value contributes positively to maintaining crop growth stability.

Second, machine learning models with SHapley Additive exPlanations (SHAP) were also employed to quantify the contributions of SOC. We applied the XGBoost model to predict crop stability with soil, climate, and management covariates, considering the same dataset as for GAMM. To run XGBoost analysis, we employed grid search coupled with 5‐fold cross‐validation to find the optimal combination of several key hyperparameters, including learning rate, max tree depth, and number of trees, by minimizing the Mean Squared Error (MSE). For each dataset of different crops, 10% of all grid cells were randomly selected for the validation set, and the remaining 90% of grid‐cells were used to train XGBoost models. To assess the goodness‐of‐fit of the constructed models, we employed four metrics: the coefficient of determination (R^2^), RMSE, normalized RMSE (nRMSE), and mean absolute percentage error (MAPE), which were commonly used in both model calibration and validation processes. The models were trained and tested using the Python package ‘xgboost’. The model accuracy can be seen in Table S4.

After the training of each XGBoost model, we used the Python package ‘shap’ to conduct SHAP analysis. The SHAP values were utilized to measure both the magnitude and direction of each feature's contribution to the model's output [47]. Thereby, we extracted the SHAP values for SOC to quantify its effect on predicted crop stability for each grid cell. Considering the differences in units of the three different productivity measures, we normalized the calculated SHAP values for better comparison as follows:

(7) $$Z_{SHAP_{SOC,i}}=\frac{SHAP_{SOC,i}}{\sum_{f}^{F}\left|SHAP_{f,i}\right|}$$

where $Z_{\mathrm{SHA}P_{\mathrm{SOC},i}}$ represents the normalized SHAP values for SOC in grid‐cell *i*; |SHAP_f,i_| represents the absolute SHAP values for feature *f* in grid‐cell *i*; F represents the total number of model features. Subsequently, we applied the locally weighted scatterplot smoothing method (LOWESS) to fit the relationship curves between SOC content and its normalized SHAP values. In this study, we divided the SOC content into sub‐intervals with a unit of 0.1 g kg^−1^, and averaged the normalized SHAP values falling within each sub‐interval. A LOWESS regression was then fitted to smooth the relationship between normalized SHAP values for SOC and baseline SOC content, considering the whole set of grid‐cells together. This nonparametric regression was fitted using the ‘statsmodels’ package in Python. Similarly, the first SOC value shifting from negative to positive SHAP values was also established as the critical SOC threshold.

#### 3.2.3 Sensitivity of Crop Productivity to Climatic Variability

To compare sensitivity of crop productivity to climate changes between regions with SOC levels below and above critical thresholds, we calculated temperature sensitivity of annual crop productivity and crop stability using two models, respectively. For computing temperature sensitivity of annual crop productivity, we used a panel data model to fit standardized annual detrended crop productivity (Δ_*R*_(*i*,*t*)) with growing‐season temperature (T,°C) and precipitation (P, mm) changes as explanatory variables:

(8) $$\Delta_{R}\;\left(i,t\right)=\alpha_{i}+\beta_{1}\;T_{i,t}+\beta_{2}T_{i,t}^{2}+\beta_{3}P_{i,t}+\beta_{4}P_{i,t}^{2}+\varepsilon_{i,t}$$

where $\Delta_{R}\;\left(i,t\right)=\frac{R_{i,t}-\mu_{R_{i}}}{\sigma_{R_{i}}}\;$, *R*_*i*,*t*_ is the detrended crop productivity (using remote sensing proxies) in year *t* at grid cell *i*. The quadratic terms of T and P simulate the nonlinear response of productivity to temperature and precipitation. β_1_ to β_4_ are regression coefficients, α_*i*_ is the grid‐cell‐specific intercept and ε_i,t_ is the model error term. Then, the temperature sensitivity can be computed as $\beta_{1}+2\beta_{2}{\bar{T}}_{i}$ where ${\bar{T}}_{i}$ is the average temperature over years in grid‐cell *i*. The closer the values are to 0, the smaller the crop productivity fluctuations driven by temperature changes. We then compare the numerical distribution and mean values of the calculated temperature sensitivity between the group including grid‐cells with SOC lower than the critical threshold and the group including grid‐cells with SOC higher than the critical threshold.

Additionally, to calculate temperature sensitivity of crop stability, we used a generalized additive model that integrated linear interaction terms with a spatial smoothing function. The model describes the crop productivity stability (*S_i_*) as:

(9) $$S_{i}=\mu +\tau_{G_{i}}+X_{i}^{T}\beta +X_{i}^{T}\left(\gamma \times l_{G_{i}}\right)+s_{tp}\left(lon_{i},lat_{i}\right)+\varepsilon_{i}$$

where μ is the global intercept, and $\tau_{G_{i}}$ represents the fixed effect of the SOC group *G_i_*. The vector *X_i_* contains the climatic variables (μ_*X*_ (mean), β_*X*_ (trend), *RMSE_X_* and *CV_X_*). β denotes the main effect of these variables, while γ captures the interaction effects between SOC groups and climatic variables, testing whether climate sensitivity differs significantly across SOC levels. $l_{G_{i}}$ is the indicator function that distinguishes SOC groups. *s_tp_* (*lon_i_*,*lat_i_*) denotes the two‐dimensional thin‐plate spline function of longitude and latitude coordinates to account for spatial autocorrelation, and ε_*i*_ is the residual error term. To mitigate group imbalance and address the large dataset size, we performed 100 bootstrap runs using subsamples of 10 000 observations from each SOC group. Temperature variability was characterized by either CV or RMSE, depending on which variable showed a stronger negative association with stability metrics across the full dataset. The resulting averaged coefficient serves as the sensitivity proxy, with more negative values signifying a higher susceptibility of crop stability to temperature variability.

#### 3.2.4 Quantifying Marginal Benefits of SOC Increase in Crop Stability From Causal Inference Models

Following the characterization of nonlinear relationships, we further aimed to advance from association to causality for quantifying the potential benefits of SOC sequestration strategies. In this study, we adopted the causal forest double machine learning model (CF‐DML) to establish causality (see detailed description of the CF‐DML model in Text S1), which was particularly well‐suited for continuous treatment variables (SOC) and captured heterogeneous treatment effects [48]. Within the CF‐DML framework, the estimated individual treatment effect (ITE) for each grid cell quantified the potential shift in crop productivity stability in response to a unit increase in SOC content. In this study, we used package ‘econml’ in Python to conduct the CF‐DML model with 100 bootstrap iterations, and defined the treatment effect of SOC on crop stability as significant for grid‐cells where more than 95% of the bootstrap estimates exhibited a positive sign. We used LOWESS regression to fit dose‐response curves between SOC content and estimated average ITE. The SOC saturation threshold was identified as the geometric knee point of the fitted curves, defined as the location of maximum deviation from the line connecting the curve's extrema. This point represented an optimal trade‐off where the marginal gain in stability begins to diminish significantly.

To rigorously validate the credibility of our causal estimates, we conducted sensitivity analysis using the ‘dowhy’ Python package. This involved three distinct refutation tests: (1) a placebo treatment test, where the treatment variable was replaced with a random variable to detect spurious correlations; (2) a random common cause test, which introduced an independent random confounder to evaluate robustness against unobserved bias; and (3) data subset validation, which involved re‐estimating the model on random data subsets to verify the consistency of the results. The results of sensitivity analyses can be seen in Table S5.

#### 3.2.5 Mechanistic Decomposition of SOC Stabilizing Pathways and Estimation of Resilience Dividend Equivalents

Before calculating the resilience dividend equivalents from SOC increase, since the stability metric in this study is defined as the negative natural logarithm of CV, we could disentangle the SOC‐driven stability marginal gains into two complementary pathways, including the enhancement of mean productivity and the reduction of interannual variability:

(10) $$S_{i}=-\ln\left(\frac{\sigma_{i}}{\mu_{i}}\right)=ln\mu_{i}-ln\sigma_{i},\;\;\Delta S_{i}=\frac{dln\mu_{i}}{dSOC}\;-\frac{dln\sigma_{i}}{dSOC}$$

where $\Delta S_{i}=\frac{dS_{i}}{dSOC}\;$ is the estimated SOC's treatment effect on stability from the CF‐DML model in grid‐cell *i*. $\frac{dln\mu_{i}}{dSOC}$ represents SOC's effect on mean productivity in gridcell *i* and $\frac{dln\sigma_{i}}{dSOC}$ represents SOC's effect on the standard deviation of productivity in grid‐cell *i*. Therefore, we built CF‐DML models targeting the natural logarithm of mean productivity, considering the same dataset as for stability, to obtain the causal effect of SOC on enhancing mean productivity. The output value was divided by Δ*S_i_* to quantify the relative contribution of the pathway to improving mean productivity in each grid‐cell. The remaining percentage was the relative contribution of the pathway to reducing standard deviation of crop productivity.

To translate stability marginal gains in each grid‐cell into tangible food security metrics, we calculated the resilience dividend equivalent, representing the amount of crop production secured by a 1 g kg^−1^ increase in SOC during a typical agricultural shock. A typical shock was defined as a year experiencing a one‐standard‐deviation negative yield loss (−1σ) [49, 50]. Statistically, this threshold corresponds to the 15.eighth percentile of yield anomalies, representing the agricultural shock that historically occurs roughly once every 6 to 7 years. We selected this benchmark to capture frequent and manageable crop failures rather than catastrophic extremes. The structural buffering capacity of SOC has absolute limits and may collapse under catastrophic events. Therefore, defining the shock at −1σ provides a conservative and reliable estimate of the resilience dividend by avoiding mathematical over‐extrapolation. The avoided yield loss was derived by integrating both the productivity raising effect and absolute fluctuation reducing effect. The calculation process is as follows:

(11) $$\begin{array}{cc} & \Delta \;Y_{c}=\left[\left(\mu_{c}+\Delta \mu_{c}\right)-1\cdot \left(\sigma_{c}+\Delta \sigma_{c}\right)\right]\;-\;\left(\mu_{c}-1\cdot \sigma_{c}\right)\\ & =\Delta \mu_{c}-\Delta \sigma_{c},\;\Delta \;\mu_{c}=\mu_{c}\;\times \Delta S_{c}\times P_{\mu_{c}},\;\Delta \;\sigma_{c}\\ & =-\sigma_{c}\times \Delta S_{c}\times P_{\sigma_{c}},\;\;\sigma_{c}=\mu_{c}\;\times e^{-S_{c}} \end{array}$$

where Δ*Y_c_* is the avoided yield loss (tonnes) attributable to a unit SOC increase in municipality *c*. μ_*c*_ is the 20‐year statistical average crop yield for municipality *c*, *S_c_* is the municipal‐level crop stability metric averaged from three remote sensing proxies for municipality *c*, and Δ*S_c_* is the municipal‐level SOC's treatment effect averaged from three remote sensing proxies for municipality *c*.$\;P_{\mu_{c}}$ is the municipal‐level percentage of SOC's effect on improving mean productivity averaged from three remote sensing proxies for municipality *c*, $P_{\sigma_{c}}$ is the municipal‐level percentage of SOC's effect on reducing standard deviation averaged from three remote sensing proxies for municipality *c*.

The municipal‐level maize and wheat annual yield and planting area data were obtained from municipal statistical yearbooks spanning 2001 to 2020 (sourced from https://data.cnki.net/).

## Author Contributions

**Yuyang Huang**: conceptualization, methodology, software, data curation, investigation, validation, formal analysis, Writing – original draft, visualization, Writing – review and editing. **Yulin Shangguan**: conceptualization, writing – review and editing, methodology, visualization. **Zhongkui Luo**: writing – review and editing, conceptualization, methodology. **Peng Zhu**: conceptualization, methodology, writing – review and editing. **David Makowski**: writing – review and editing, methodology. **Zhenzhong Zeng**: writing – review and editing, methodology. **Qingxu Ma**: conceptualization, writing – review and editing. **Min Wei**: investigation. **Yuanyuan Du**: investigation. **Su Ye**: conceptualization, methodology. **Zhou Shi**: conceptualization, methodology, supervision, project administration, resources, funding acquisition, visualization, writing – review and editing. **Songchao Chen**: writing – review and editing, supervision, resources, project administration, funding acquisition, conceptualization, methodology, visualization.

## Conflicts of Interest

The authors declare no conflicts of interest.

## Code Availability Statement

The scripts used for data analysis are available at https://doi.org/10.5281/zenodo.18982061.

## Supporting information




**Supporting File**: advs78421‐sup‐0001‐SuppMat.docx.

## Data Availability

The data used in this study are available at https://doi.org/10.5281/zenodo.18982061.
