# Supplementary material for: Enriched Soil Organic Carbon Underpins Long‐Term Stable Crop Productivity Across China's Croplands
Source: Adv Sci (Weinh). 2026 Oct 22:e78421. Online ahead of print. doi: 10.1002/advs.78421 (PMC13691224; doi:10.1002/advs.78421)
Supplement: Supplementary file 1 [file ADVS-9999-e78421-s001.docx]

Supplementary Materials for

**Enriched soil organic carbon underpins long-term stable crop productivity across China's croplands**

Yuyang Huang *et al.*

*Corresponding author. Email: chensongchao@zju.edu.cn

**This file includes:**

Supplementary Text

Figs. S1 to S10

Tables S1 to S10

References (1 to 10)

Supplementary Text

Introduction to causal forest double machine learning model.

In this study, we adopted the causal forest double machine learning model (CF-DML) to establish causality. The double machine learning model aims to minimize biases from confounding variables and provide more reliable causal effect estimates with two-step process: (1) estimating the outcome and treatment variables with control covariates using machine learning models and calculating residuals to remove the effects of control covariates on the outcome and treatment variables (Chernozhukov et al., 2017; Guo et al., 2024):

where Y denotes the outcome variable; D denotes the treatment variable; Z denotes the control covariates;indicates model’s unobserved factors (captured by and, respectively) are not systematically related to the covariates X. (2) estimating the causal treatment effects from residuals of the outcome and treatment variables:

where and are the predicted outcome and treatment variables, respectively;represents the causal effect value of D on Y; is the error term. There are numerous methods to estimate the causal parameter, including linear regression and nonparametric functions.

Here, we employed the causal forest model to estimate the causal parameter. This approach is particularly well-suited for continuous treatment variables (SOC content) and is specifically designed to capture heterogeneous treatment effects (Athey and Wager, 2019). A defining feature of this model is the implementation of “honest trees” to ensure valid statistical inference. Specially, for each tree, a random subset of size $s$ is procured from original dataset and partitioned into two independent halves: splitting samples used to determine the tree structure, and estimation samples used to evaluate treatment effects within the leaf nodes (Athey and Wager, 2019). Based on the minimization objective in Equation (2), the conditional average treatment effects (CATE) for a specific leaf node $L$ is estimated as:

where represents the estimated CATE for the leaf node L; represents the estimation samples belonging to the leaf node L.

To construct the causal trees, the splitting criterion is designed to maximize the heterogeneity of treatment effects across leaf nodes. Specially, the causal tree recursively partitions the data to maximize the squared difference in CATE between the two resulting child nodes ( C_1_ and C_2_), weighted by their respective sample sizes ( and) (Guo et al., 2024):

Therefore, for causal forests consisting of $K$ distinct causal trees, the individual treatment effect (ITE) of each entity which is defined by Z = z can be calculated as:

Within the CF-DML framework, the estimated ITE for each pixel quantifies the potential shift in crop productivity stability in response to a unit increase in SOC content. Consequently, this metric serves as a direct indicator of the marginal causal effect of SOC, specifically characterizing its efficacy under the unique environmental context of each observation.

Fig. S1. Spatial patterns of crop productivity stability derived from multiple productivity proxies. a - c, Stability of maize productivity calculated using NIRv (a), SIF (b), and MODIS GPP (c). d-f, Stability of wheat productivity calculated using NIRv (d), SIF (e), and MODIS GPP (f). Although the absolute magnitudes of stability metrics vary across proxies, their spatial distributions remain consistent.

Fig. S2. Distribution of SOC content in cropland cultivated with maize (a) and wheat (b).

Fig. S3. Treatment effect of SOC on enhancing the stability of maize and wheat productivity (using NIRv and MODIS GPP as proxies) as estimated by the CausalForestDML model. a,b, the spatial distribution of treatment effect of SOC on enhancing the stability of maize productivity using NIRv (a) and MODIS GPP (b) as proxies. c,d, the spatial distribution of treatment effect of SOC on enhancing the stability of wheat productivity using NIRv (c) and MODIS GPP (d) as proxies. The bar charts illustrate the number of grid-cells within each grading of SOC treatment effect values, and the pie charts display the proportion of grid-cells with a significant SOC effect and their distribution across different SOC levels.

Fig. S4. Correlation rankings between covariates and treatment effect of SOC. Triangles indicate the primary environmental drivers (from two distinct categories, matching Fig. 4) that show the highest correlation with SOC treatment effects, utilized here to assess marginal stability benefits under different environmental contexts.

**Fig. S5. Sensitivity analysis of the SOC-crop stability relationships and the SOC stabilizing effect evaluated at different spatial resolutions.** All results obtained from NIRv values across China’s maize cropland. a, relationships between baseline SOC content and the stability of maize productivity obtained from GAMM models. b, relationships between SOC content and its SHAP values obtained from XGBoost models. c, boxplots of national treatment effect obtained from CF-DML models. d, boxplots of treatment effect in significant grid-cells.

**Fig. S6. Crop productivity sensitivity to temperature and crop stability sensitivity to temperature variability in low SOC and high SOC regions distinguished by the lower bound of critical threshold ranges (10.5 g kg⁻¹ for maize, and 8.4 g kg⁻¹ for wheat).**

**Fig. S7. Crop productivity sensitivity to temperature and crop stability sensitivity to temperature variability in low SOC and high SOC regions distinguished by the upper bound of critical threshold ranges (13.1 g kg⁻¹ for maize, and 9.9 g kg⁻¹ for wheat).**

**Fig. S8. The relationships between SOC baselines and the stability of maize productivity across different major agricultural zones in China.** NCP: Northeast China Plain, NASR: Northern arid and semiarid region, HHHP: Huang-Huai-Hai Plain, LP: Loess Plateau, SBSR: Sichuan Basin and surrounding regions, YGP: Yunnan-Guizhou Plateau, QTP: Qinghai Tibet Plateau, MLYP: Middle-lower Yangtze Plain, SC: Southern China. Only agricultural regions possessing an adequate number of valid pixels (highlighted in red) were selected for analysis. Regional sub-datasets were subsequently utilized to develop GAMMs, aiming to quantify the relationship between SOC baselines and crop productivity stability and to identify the critical SOC threshold ranges specific to each agricultural region.

**Fig. S9. The relationships between SOC baselines and the stability of wheat productivity across different major agricultural zones in China.** NCP: Northeast China Plain, NASR: Northern arid and semiarid region, HHHP: Huang-Huai-Hai Plain, LP: Loess Plateau, SBSR: Sichuan Basin and surrounding regions, YGP: Yunnan-Guizhou Plateau, QTP: Qinghai Tibet Plateau, MLYP: Middle-lower Yangtze Plain, SC: Southern China. Only agricultural regions possessing an adequate number of valid pixels (highlighted in red) were selected for analysis. Regional sub-datasets were subsequently utilized to develop GAMMs, aiming to quantify the relationship between SOC baselines and crop productivity stability and to identify the critical SOC threshold ranges specific to each agricultural region.

**Fig. S10. The relationships between SOC content and the stability of maize and wheat yield based on long-term site-scale measured crop yield and SOC data.** This long-term experimental dataset comprises in-situ measurements from 22 maize and 20 wheat monitoring sites across China. For each site, the dataset includes over a decade of annual crop yield records under various field management practices, coupled with corresponding SOC measurements recorded at 5-year intervals. Adopting the identical methodology from our primary analysis, we calculated the crop yield stability metrics and mean SOC levels for each management practice at every site. Simultaneously, auxiliary climate, soil, and management covariates were extracted based on the geographical coordinates of the sites, culminating in a new in-situ dataset. By applying the GAMM model used in the main analysis, we quantified the partial relationship between SOC content and crop yield stability. This framework yielded critical SOC thresholds of 9.3 g kg⁻¹ for maize and 10.5 g kg⁻¹ for wheat.

Table S1. Statistics of crop productivity stability metrics derived from multiple productivity metrics. Note: P5, P25, P75, and P95 denote the 5th, 25th, 75th, and 95th percentiles, respectively.

| Crop Type | Proxy | P5 | P25 | Median | P75 | P95 | Average |
| --- | --- | --- | --- | --- | --- | --- | --- |
| Maize | NIRv | -2.95 | -2.62 | -2.40 | -2.17 | -1.86 | -2.40 |
|  | SIF | -2.64 | -2.33 | -2.12 | -1.91 | -1.58 | -2.12 |
|  | MODIS GPP | -2.76 | -2.53 | -2.34 | -2.11 | -1.84 | -2.32 |
| Wheat | NIRv | -3.31 | -2.92 | -2.73 | -2.55 | -2.26 | -2.75 |
|  | SIF | -3.04 | -2.71 | -2.52 | -2.36 | -2.05 | -2.53 |
|  | MODIS GPP | -2.98 | -2.62 | -2.45 | -2.32 | -2.12 | -2.49 |

Table S2. Covariates for associational and causal models.

| Data Type | Covariates | Abbreviation | Unit | Resolution | Data Source |
| --- | --- | --- | --- | --- | --- |
| Climatic variables | Average  temperature | Tmp | ℃ | 1km | Qin et al., 2022 |
|  | Average total precipitation | Prep | mm | 1km | Qin et al., 2022 |
|  | CV of annual average temperature | CV_Tmp | % | 1km | Qin et al., 2022 |
|  | CV of annual total precipitation | CV_Prep | % | 1km | Qin et al., 2022 |
|  | Annual trends of mean temperature | Tmp_trend | / | 1km | Qin et al., 2022 |
|  | Annual trends of total precipitation | Prep_trend | / | 1km | Qin et al., 2022 |
|  | Residuals of temperature trends | Tmp_RMSE | / | 1km | Qin et al., 2022 |
|  | Residuals of precipitation trends | Prep_RMSE | / | 1km | Qin et al., 2022 |
| Soil variables | Soil organic carbon | SOC | g/kg | 250m | Liu et al., 2022 |
|  | Soil bulk density | BD | g/cm^3^ | 250m | Liu et al., 2022 |
|  | Soil pH | pH | / | 250m | Liu et al., 2022 |
|  | Clay content | Clay | g/kg | 250m | Liu et al., 2022 |
|  | Silt content | Silt | g/kg | 250m | Liu et al., 2022 |
|  | Sand content | Sand | g/kg | 250m | Liu et al., 2022 |
|  | Soil cation exchange capacity | CEC | cmol(+)/kg | 250m | Liu et al., 2022 |
| Management variables | Nitrogen  fertilizer use | Nfer | gN/m^2^‧yr | 5km | Yu et al., 2022 |
|  | Phosphorus fertilizer use | Pfer | gP_2_O_5_/m^2^‧yr | 1km | Zhang et al., 2023 |
|  | Fraction of irrigated cropland | Irri_fraction | % | 250m | Zhang et al., 2024 |
|  | Irrigation water use | Irri_IWU | mm/yr | 500m | Bo et al., 2026 |
|  | Changes in planting density | Plantdensity | yr^-1^ | 500m | MOD15A2H LAI product |
|  | Tillage practices | Tillage | / | 5 arc-minutes | Porwollik et al., 2019 |

Note: All covariates are resampled to 250m to match with the spatial resolution of crop productivity stability.

Table S3. Accuracy of GAMM models for fitting crop productivity stability. Note: R^2^ (adjusted): the proportion of variance explained by the model. Deviance explained (%): the percentage of null deviance explained by the model, serving as a measure of goodness-of-fit. SOC edf: estimated degrees of freedom for the SOC smooth term, reflecting the degree of non-linearity in the relationship between SOC content and crop productivity stability. An edf of 1 indicates a strictly linear relationship, whereas an edf > 1 indicates a non-linear relationship, with larger values signifying higher fluctuation and complexity of the non-linear curve. SOC F-value: the F-statistic for the SOC smooth term, reflecting the relative importance of SOC in explaining the variance of the dependent variable within the model. SOC p-value: the statistical significance level of the SOC effect.

| Crop Type | Proxy | R^2^ (adjusted) | Deviance explained (%) | SOC edf | SOC  F-value | SOC  p-value |
| --- | --- | --- | --- | --- | --- | --- |
| Maize | NIRv | 0.397 | 39.7 | 17.925 | 7708.7 | <0.001^***^ |
|  | SIF | 0.470 | 47.0 | 14.799 | 8066.0 | <0.001^***^ |
|  | MODIS GPP | 0.608 | 60.8 | 14.803 | 6088.0 | <0.001^***^ |
| Wheat | NIRv | 0.359 | 35.9 | 5.361 | 4192.38 | <0.001^***^ |
|  | SIF | 0.434 | 43.5 | 5.346 | 4319.2 | <0.001^***^ |
|  | MODIS GPP | 0.423 | 42.3 | 4.239 | 8770.97 | <0.001^***^ |

Table S4. Accuracy of XGBoost models for fitting crop productivity stability.

| Crop Type | | Maize | | | Wheat | | |
| --- | --- | --- | --- | --- | --- | --- | --- |
| Proxy | | NIRv | SIF | MODIS GPP | NIRv | SIF | MODIS GPP |
| Training Set | R^2^ | 0.605 | 0.689 | 0.756 | 0.634 | 0.762 | 0.734 |
|  | RMSE | 0.204 | 0.168 | 0.139 | 0.184 | 0.135 | 0.131 |
|  | nRMSE (%) | 8.543 | 7.908 | 6.027 | 6.676 | 5.330 | 5.294 |
|  | MAPE (%) | 6.680 | 6.384 | 4.810 | 5.150 | 4.017 | 4.068 |
| Validation Set | R^2^ | 0.598 | 0.683 | 0.752 | 0.617 | 0.748 | 0.719 |
|  | RMSE | 0.206 | 0.170 | 0.141 | 0.188 | 0.138 | 0.135 |
|  | nRMSE (%) | 8.623 | 7.970 | 6.080 | 6.841 | 5.459 | 5.436 |
|  | MAPE (%) | 6.733 | 6.426 | 4.848 | 5.266 | 4.100 | 4.159 |

Table S5. Sensitivity analyses and robustness checks for causal forest double machine learning models. Note: (1) Placebo Treatment Test: The treatment variable was replaced with a random placebo. A Placebo ATE close to 0 with a high p-value (> 0.05) indicates that the estimated effect is not driven by spurious correlations. (2) Random Common Cause Test: An independent random variable was added as a confounder. A New effect close to the original Estimated effect with a high p-value implies the result is insensitive to unobserved random noise. (3) Data Subset Validation: The model was re-estimated using a random subset of the data. Consistency between the new effect and the original estimate, supported by a high p-value, suggests the causal effect is robust to sample selection.

| Crop Type | | | Maize | | | Wheat | | |
| --- | --- | --- | --- | --- | --- | --- | --- | --- |
| Proxy | | | NIRv | SIF | MODIS GPP | NIRv | SIF | MODIS GPP |
| Refutation Tests | Placebo Treatment Test | Placebo ATE | 0.000 | 0.000 | 0.000 | 0.000 | 0.000 | 0.000 |
|  |  | p-value | 0.988 | 0.978 | 0.983 | 0.989 | 0.962 | 0.947 |
|  | Random Common Cause Test | Estimated effect | 0.022 | 0.014 | 0.013 | 0.021 | 0.019 | 0.019 |
|  |  | New effect | 0.021 | 0.015 | 0.013 | 0.022 | 0.018 | 0.019 |
|  |  | p-value | 0.460 | 0.280 | 0.680 | 0.170 | 0.260 | 0.700 |
|  | Data Subset Validation | Estimated effect | 0.022 | 0.014 | 0.013 | 0.021 | 0.019 | 0.019 |
|  |  | New effect | 0.021 | 0.015 | 0.013 | 0.022 | 0.018 | 0.018 |
|  |  | p-value | 0.460 | 0.52 | 0.500 | 0.460 | 0.520 | 0.680 |

Table S6. Statistics of sensitivity of productivity to annual mean temperature across three productivity proxies in low and high SOC zones. Note: P25 and P75 denote the 25th and 75th percentiles, respectively.

| Crop Type | Proxy | Region | P25 (%) | Mean (%) | P75 (%) |
| --- | --- | --- | --- | --- | --- |
| Maize | NIRv | Low SOC | -40.02 | -18.61 | 0.79 |
|  |  | High SOC | -21.31 | 1.31 | 22.35 |
|  | SIF | Low SOC | -37.61 | -19.39 | -3.00 |
|  |  | High SOC | -23.22 | -2.28 | 16.36 |
|  | MODIS GPP | Low SOC | -55.86 | -33.86 | -16.04 |
|  |  | High SOC | -18.48 | 2.88 | 26.06 |
| Wheat | NIRv | Low SOC | -18.51 | 9.84 | 38.85 |
|  |  | High SOC | -22.28 | 3.61 | 28.42 |
|  | SIF | Low SOC | -7.41 | 19.95 | 48.29 |
|  |  | High SOC | -25.07 | 1.88 | 27.86 |
|  | MODIS GPP | Low SOC | 32.00 | 42.78 | 62.62 |
|  |  | High SOC | -3.07 | 29.85 | 63.74 |

Table S7. Statistics of average treatment effect of SOC and corresponding changes in coefficient of variation of crop productivity obtained from CF-DML models. Note: ATE denotes the national average treatment effect of SOC, ATE (sig.) denotes the average treatment effect in grid-cells with significant effect. -ΔCV is calculated from the average treatment effect based on stability metric, and also represents the stability gains in the study.

| Crop Type | Proxy | ATE | ATE (sig.) | -ΔCV  (stability gains) | -ΔCV (sig.) |
| --- | --- | --- | --- | --- | --- |
| Maize | NIRv | 0.020 | 0.068 | 1.97% | 6.54% |
|  | SIF | 0.014 | 0.057 | 1.39% | 5.53% |
|  | MODIS GPP | 0.011 | 0.047 | 1.11% | 4.59% |
| Wheat | NIRv | 0.018 | 0.096 | 1.77% | 9.15% |
|  | SIF | 0.015 | 0.077 | 1.50% | 7.43% |
|  | MODIS GPP | 0.015 | 0.077 | 1.52% | 7.41% |

Table S8. Statistics of average treatment effect of SOC and corresponding changes in coefficient of variation of crop productivity obtained from CF-DML models excluding management variables.

| Crop Type | Proxy | ATE | ATE (sig.) | -ΔCV  (stability gains) | -ΔCV (sig.) |
| --- | --- | --- | --- | --- | --- |
| Maize | NIRv | 0.020 | 0.089 | 1.99% | 8.50% |
|  | SIF | 0.018 | 0.077 | 1.82% | 7.41% |
|  | MODIS GPP | 0.011 | 0.061 | 1.14% | 5.93% |
| Wheat | NIRv | 0.013 | 0.099 | 1.33% | 11.81% |
|  | SIF | 0.021 | 0.102 | 2.06% | 9.67% |
|  | MODIS GPP | 0.013 | 0.100 | 1.26% | 9.54% |

**Table S9. Sensitivity analysis of the spatial coverage of SOC functional phases to the selection of critical threshold boundaries.** Note: the upper bound represents the inclusive boundary adopted to distinguish the high-gains phase (maize 13.1 g kg⁻¹; Wheat: 9.9 g kg⁻¹), whereas the lower bound serves as a conservative sensitivity test (use maize 10.5 g kg⁻¹; Wheat: 8.4 g kg⁻¹). Regardless of the boundaries applied, the spatial extent of the high-gains phase consistently accounts for over 15% of the national crop area across all three productivity proxies.

| Crop | | Maize | | | Wheat | | |
| --- | --- | --- | --- | --- | --- | --- | --- |
| Percentage | | NIRv | SIF | MODIS GPP | NIRv | SIF | MODIS GPP |
| Upper bound | High gains Phase | 22.0% | 19.4% | 19.8% | 23.2% | 22.7% | 22.6% |
|  | Diminishing effect Phase | 9.7% | 7.9% | 7.3% | 3.1% | 2.9% | 2.7% |
|  | Saturation Phase | 1.4% | 1.3% | 1.1% | 1.0% | 1.1% | 1.0% |
|  | No Sig. | 66.9% | 71.4% | 71.8% | 72.7% | 73.3% | 73.7% |
| Lower bound | High gains Phase | 19.5% | 17.6% | 17.7% | 14.9% | 15.5% | 15.6% |
|  | Diminishing effect Phase | 12.2% | 9.7% | 9.4% | 11.5% | 10.1% | 9.7% |
|  | Saturation Phase | 1.4% | 1.3% | 1.1% | 9.9% | 1.1% | 1.0% |
|  | No Sig. | 66.9% | 71.4% | 71.8% | 72.7% | 73.3% | 73.7% |

Table S10. Statistical information of identified high-gains municipalities. Note: Ratio denotes the percentage of high-gains grid-cells coverage in the city. Stability is the city-level average of the stability metric. Yield is the 20-year average yield recorded in the statistical yearbooks. Yield_saved represents the estimated averted yield loss during agricultural shocks from a unit increase in SOC. Q indicates which quadrant the city is located in (1: ‘high yield and high stability’; 2: ‘low yield and high stability’; 3: ‘low yield and low stability’; 4: ‘high yield and low stability’.)

| Crop Type | Province | City | Ratio | Stability | Yield (tonnes/ha) | Yield_saved (tonnes) | Q |
| --- | --- | --- | --- | --- | --- | --- | --- |
| Wheat | Hebei | Cangzhou | 0.55 | -2.86 | 5.29 | 120231.3 | 4 |
|  | Hebei | Handan | 0.27 | -2.79 | 6.63 | 81237.11 | 4 |
|  | Hebei | Hengshui | 0.47 | -2.85 | 5.25 | 90026.68 | 4 |
|  | Hebei | Langfang | 0.20 | -2.76 | 8.76 | 3352.682 | 4 |
|  | Hebei | Shijiazhuang | 0.21 | -2.71 | 7.26 | 53688.4 | 4 |
|  | Hebei | Tangshan | 0.16 | -2.6 | 5.6 | 12357.76 | 4 |
|  | Hebei | Xingtai | 0.37 | -2.88 | 6.15 | 92540.74 | 4 |
|  | Henan | Anyang | 0.16 | -2.55 | 6.23 | 26896.61 | 1 |
|  | Henan | Jiaozuo | 0.42 | -2.64 | 6.93 | 40109.54 | 4 |
|  | Henan | Luoyang | 0.49 | -2.77 | 4.59 | 36919.53 | 3 |
|  | Henan | Pingdingshan | 0.37 | -2.59 | 4.55 | 27995.85 | 3 |
|  | Henan | Xinxiang | 0.18 | -2.59 | 5.66 | 21692.76 | 4 |
|  | Henan | Zhenzhou | 0.41 | -2.66 | 5.51 | 21997.89 | 4 |
|  | Shaanxi | Xian | 0.20 | -2.6 | 6.3 | 15032.79 | 4 |
|  | Shandong | Binzhou | 0.33 | -2.74 | 5.08 | 48881.68 | 4 |
|  | Shandong | Dezhou | 0.39 | -2.65 | 5.58 | 147983.6 | 4 |
|  | Shandong | Jinan | 0.25 | -2.55 | 5.56 | 30807.99 | 1 |
|  | Shandong | Jining | 0.22 | -2.54 | 6 | 64888.75 | 1 |
|  | Shandong | Liaocheng | 0.39 | -2.58 | 5.85 | 105041.4 | 4 |
|  | Shandong | Linyi | 0.18 | -2.56 | 6.07 | 35799.52 | 1 |
|  | Shandong | Qingdao | 0.21 | -2.61 | 5.88 | 47093.14 | 4 |
|  | Shandong | Taian | 0.24 | -2.52 | 7.14 | 60060.43 | 1 |
|  | Shandong | Weifang | 0.23 | -2.76 | 6.29 | 105342.1 | 4 |
|  | Shandong | Yantai | 0.19 | -2.51 | 7.03 | 54088.29 | 1 |
|  | Shandong | Zaozhuang | 0.24 | -2.58 | 6.11 | 23272.79 | 4 |
|  | Shandong | Zibo | 0.25 | -2.64 | 6.81 | 24823.51 | 4 |
|  | Shanxi | Jinzhong | 0.29 | -2.81 | 3.62 | 4511.062 | 3 |
|  | Shanxi | Yuncheng | 0.41 | -2.91 | 3.77 | 34772.41 | 3 |
| Average | / | / | 0.30 | -2.67 | 5.91 | 51123.08 | / |
| Maize | Gansu | Dingxi | 0.89 | -2.57 | 4.28 | 14362.52 | 3 |
|  | Gansu | Jincang | 0.63 | -2.45 | 4.41 | 5034.468 | 3 |
|  | Gansu | Jiuquan | 0.60 | -2.14 | 5.92 | 9917.085 | 1 |
|  | Gansu | Linxia | 0.88 | -2.38 | 5.53 | 15041.3 | 4 |
|  | Gansu | Pingliang | 0.83 | -2.51 | 4.64 | 13693.2 | 3 |
|  | Gansu | Zhangye | 0.68 | -2.14 | 5.67 | 26905.41 | 1 |
|  | Hebei | Hengshui | 0.43 | -2.36 | 4.67 | 34930.41 | 3 |
|  | Hebei | Zhangjiakou | 0.71 | -2.45 | 4.11 | 43654.54 | 3 |
|  | Liaoning | Chaoyang | 0.68 | -2.09 | 4.25 | 40170.6 | 2 |
|  | Liaoning | Fuxin | 0.67 | -2.14 | 4.84 | 41682.14 | 1 |
|  | Neimenggu | Baotou | 0.46 | -2.36 | 5.96 | 37736.9 | 4 |
|  | Neimenggu | Bayanzhuoer | 0.39 | -2.19 | 4.77 | 49159.95 | 2 |
|  | Neimenggu | Chifeng | 0.68 | -2.23 | 5.17 | 119412.1 | 1 |
|  | Neimenggu | Eerduosi | 0.63 | -2.24 | 4.1 | 87538.83 | 2 |
|  | Neimenggu | Huhehaote | 0.58 | -2.5 | 3.81 | 36871.46 | 3 |
|  | Neimenggu | Wulancabu | 0.58 | -2.74 | 3.08 | 11071.97 | 3 |
|  | Ningxia | Guyuan | 0.90 | -2.66 | 3.2 | 9456.422 | 3 |
|  | Ningxia | Wuzhong | 0.54 | -2.48 | 6 | 16783.13 | 4 |
|  | Ningxia | Zhongwei | 0.79 | -2.35 | 5.26 | 19997.19 | 4 |
|  | Shaanxi | Ankang | 0.82 | -2.07 | 3.16 | 15120.68 | 2 |
|  | Shaanxi | Shangluo | 0.70 | -2 | 4.13 | 6404.649 | 2 |
|  | Shanxi | Changzhi | 0.71 | -2.44 | 5.59 | 27931.99 | 4 |
|  | Shanxi | Datong | 0.73 | -2.42 | 4.45 | 35726.12 | 3 |
|  | Shanxi | Linfen | 0.45 | -2.6 | 3.8 | 18447.33 | 3 |
|  | Shanxi | Lvliang | 0.76 | -2.36 | 4 | 20311.32 | 3 |
|  | Shanxi | Suzhou | 0.75 | -2.36 | 4.71 | 57531.81 | 3 |
|  | Shanxi | Taiyuan | 0.66 | -2.29 | 6.77 | 7883.212 | 4 |
|  | Shanxi | Xinzhou | 0.71 | -2.27 | 4.83 | 44691.08 | 1 |
|  | Shanxi | Yangquan | 0.92 | -2.08 | 4.89 | 12638.94 | 1 |
|  | Xinjiang | Cangji | 0.69 | -2.46 | 12.77 | 33069.63 | 4 |
| Average | / | / | 0.68 | -2.34 | 4.96 | 30439.21 | / |

References

Athey, S., Wager, S., 2019. Estimating Treatment Effects with Causal Forests: An Application. https://doi.org/10.48550/arXiv.1902.07409

Bo, Y., Li, X., Liu, K., Wang, S., Li, L., Li, G., Li, H., 2026. Spatially explicit estimation of high-resolution irrigation water use across China using earth observation data and deep learning. ISPRS Journal of Photogrammetry and Remote Sensing 237, 514–544. https://doi.org/10.1016/j.isprsjprs.2026.05.002

Chernozhukov, V., Chetverikov, D., Demirer, M., Duflo, E., Hansen, C., Newey, W., 2017. Double/Debiased/Neyman Machine Learning of Treatment Effects. American Economic Review 107, 261–265. https://doi.org/10.1257/aer.p20171038

Guo, Y., Li, M., Li, K., Li, H., Li, Y., 2024. Unraveling the determinants of traffic incident duration: A causal investigation using the framework of causal forests with debiased machine learning. Accident Analysis & Prevention 208, 107806. https://doi.org/10.1016/j.aap.2024.107806

Liu, F., Wu, H., Zhao, Y., Li, D., Yang, J.-L., Song, X., Shi, Z., Zhu, A.-X., Zhang, G.-L., 2022. Mapping high resolution National Soil Information Grids of China. Science Bulletin 67, 328–340. https://doi.org/10.1016/j.scib.2021.10.013

Porwollik, V., Rolinski, S., Heinke, J., Müller, C., 2019. Generating a rule-based global gridded tillage dataset. Earth System Science Data 11, 823–843. https://doi.org/10.5194/essd-11-823-2019

Qin, R., Zhao, Z., Xu, J., Ye, J.-S., Li, F.-M., Zhang, F., 2022. HRLT: a high-resolution (1d, 1km) and long-term (1961–2019) gridded dataset for surface temperature and precipitation across China. Earth System Science Data 14, 4793–4810. https://doi.org/10.5194/essd-14-4793-2022

Yu, Z., Liu, J., Kattel, G., 2022. Historical nitrogen fertilizer use in China from 1952 to 2018. Earth System Science Data 14, 5179–5194. https://doi.org/10.5194/essd-14-5179-2022

Zhang, L., Xie, Y., Zhu, X., Ma, Q., Brocca, L., 2024. CIrrMap250: annual maps of China’s irrigated cropland from 2000 to 2020 developed through multisource data integration. Earth System Science Data 16, 5207–5226. https://doi.org/10.5194/essd-16-5207-2024

Zhang, W., Zhang, T., Yang, X., 2023. 1 km-resolution gridded dataset of phosphorus rate for rice wheat and maize in China over 2004–2016. Sci Data 10, 363. https://doi.org/10.1038/s41597-023-02283-z
